# MCP-1 is overexpressed in triple-negative breast cancers and drives cancer invasiveness and metastasis

**DOI:** 10.1007/s10549-018-4760-8

**Published:** 2018-03-28

**Authors:** Pranabananda Dutta, Marianna Sarkissyan, Kimberly Paico, Yanyuan Wu, Jaydutt V. Vadgama

**Affiliations:** 1 Division of Cancer Research and Training, Department of Medicine, Charles R. Drew University of Medicine and Science, 1731 East 120th Street, Los Angeles, CA 90059 USA; 2 Jonsson Comprehensive Cancer Center, David Geffen School of Medicine, University of California at Los Angeles, Los Angeles, CA USA

**Keywords:** MCP-1, TNBC, Metastasis, MAP kinase

## Abstract

**Background:**

Triple-negative breast cancer (TNBC) is the most aggressive type of breast cancer that lacks ER/PR and HER2 receptors. Hence, there is urgency in developing new or novel therapeutic strategies for treatment of TNBC. Our study shows that the Monocyte Chemoattractant Protein-1 (MCP-1) is a marker associated with TNBC and may play a key role in TNBC disease progression.

**Experimental design:**

ELISA method was used to measure secreted MCP-1, and mRNA levels were determined by Real-time PCR in numerous cancer cell lines, representing various breast cancer subtypes. Cellular invasiveness was determined by Boyden chamber assay.

**Results:**

Our data show that MCP-1 is upregulated in TNBC cell lines both transcriptionally as well as in secreted protein levels compared to ER-positive luminal cell line, MCF-7. Breast cancer patients, with Basal or Claudin-low subtypes, also showed high expression of MCP-1. MCP-1 treatment induced cell invasion in various breast cancer cell types, without affecting cell proliferation. Small molecule antagonists against Chemokine Receptor 2 (CCR2), cognate receptor for MCP-1 as well as the MAP kinase pathway inhibitor U0126 negatively affected MCP-1 induced MCF-7 cell invasion. This suggests that MCP-1-CCR2 axis may regulate invasiveness via the MAP Kinase pathway. Knocking down MCP-1 decreased cell invasion in TNBC cell line BT-549, along with downregulation of key epithelial to mesenchymal transition markers, N-cadherin and Vimentin.

**Conclusion:**

Our study suggests that MCP-1 mediated pathways could be potential therapeutic targets for the treatment of TNBC, and could reduce cancer health disparities.

**Electronic supplementary material:**

The online version of this article (10.1007/s10549-018-4760-8) contains supplementary material, which is available to authorized users.

## Introduction

Breast cancer is the second leading cause of mortality in women. According to SEER (surveillance, epidemiology, and end result program: seer.cancer.gov), more than 200 thousand new breast cancer cases were diagnosed in 2016 in the United States. The estimated death remains to be more than 40 thousand cases in the same year. Between the various histological subtypes of breast cancer, Triple-negative breast cancer (TNBC) comprises only 15–20% of total breast cancer cases. However, thus far, TNBC is difficult to treat due to the lack of any targeted therapy [1, 2]. TNBCs do not express estrogen (ER) or progesterone (PR) receptors or HER2 and lack specific biomarkers that could be correlated with disease incidence and progression [3]. TNBCs represent a significant health disparity as these occur at higher rates in African-American (AA) women compared to European-American women. TNBCs are also diagnosed at younger ages (< 40 years) [4]. Thus far, the biological factors responsible for this health disparity in TNBC are unknown. Therefore, understanding the cellular or molecular nature of this health disparity is critical so that we can develop better treatment options for this type of breast cancer. Obesity is a well-known comorbidity in breast cancer and could contribute to this disparity as well. Obese African-American breast cancer patients have been shown to have lower disease-free survival compared with nonobese counterparts [5]. Obesity-related inflammation has been shown to involve crosstalk between cancer cells and its microenvironment via chemokines that cause metastasis [6]. Monocyte Chemoattractant protein 1 (MCP-1) is one such inflammatory chemokine implicated in cancer development and progression [7]. MCP-1 is a member of the C–C motif chemokine family. It is encoded from a locus at human Chromosome 17 and comprises of 76 amino acids [8]. It is a 12kd protein that is secreted from the cell and binds to its receptor CCR2. The receptor for MCP-1 is a G-protein coupled receptor that activates downstream components upon ligand binding.

We have previously observed that MCP-1, along with GROα, serum levels were high in obese AA breast cancer patients compared with nonobese patients (unpublished observation). Cytokines like GROα was found to be highly expressed in TNBC and was associated with metastasis [9]. In our current study, we show that higher MCP-1 levels are also associated with cell invasion and metastasis in breast cancer. Here we demonstrate that, MCP-1 is highly expressed in breast cancer cells. Importantly, the expression levels in TNBC tumor cells are higher compared with other subtypes of breast cancer cells. Although, MCP-1 modestly affects cellular proliferation, it is a significant driver of cellular invasiveness in breast cancer. Our data shows MCP-1 regulating cell invasiveness in breast cancer via p44/42 MAP kinase (MAPK) pathway. Notably, our work indicates that MCP-1 is a potential biomarker for TNBC.

## Materials and methods

### Cell cultures, antibodies, and reagents

All cell lines were purchased from the ATCC (American type culture collection). MDA-MB-231, MCF-7, SK-BR-3, BT549, and T47D cells were grown in DMEM/F12 medium containing 10% FBS, 1 mM Glutamine, and antibiotics (Penicillin/Streptomycin) in 5% CO2 incubator at 37 °C. HCC70, HCC1937, HCC1806, and HCC1395 were cultured similarly except when RPMI-1640 was used.

### ELISA for human MCP-1

ELISA was performed using the human CCL2/MCP-1 Quantikine ELISA Kit (R&D Biosystems DCP00). Equal number of cells per condition was plated for overnight and then incubated with serum-free “Optimem” media for additional 24 h. The condition media was collected for measuring secreted MCP-1 protein level. MCP-1 levels (ng/ml) were normalized to 1 × 10^6^ cells. The assay was independently performed twice and the level of MCP-1 in each cell line was mean from the two times’ measurements plus standard deviation.

### RNA isolation and quantitative real-time PCR

RNA was extracted from sub-confluent cells with RNA Bee (Tel-Test) and cDNA synthesis was performed using Quantabio cDNA synthesis master mix (95048) following manufacturer instruction. The expression levels of a MCP-1 and MMP9 were tested along with an 18S control by qPCR with specific primers (supplement methods) in triplicates. The results were analyzed by the 2−ΔΔCt method to show relative expression over 18S as control gene. The RT-qPCR was performed twice with RNA extracted from two independent sub-confluent or treated cells for each experiment.

### Boyden chamber invasion assay

8-μM pore 24-well Boyden chamber inserts were coated with Matrigel (BD Biosciences), and cells were plated on the top chamber. The bottom chamber contained serum-free medium with the indicated amount of recombinant human MCP-1(rhMCP-1). When CCR2 antagonist and MEK inhibitors (U0126) were used, the cells were plated with the inhibitor on the top chambers. Each condition was plated in duplicates, and the assays were performed twice for each cell line. The invaded cells were counted using the 20X objective of a Leica DM IRB inverted microscope from three randomly selected fields for each condition after 38 to 48 h as indicated in figure legends. The results are presented as the mean number of cells per field for each condition.

### Western blotting

Total protein was extracted, and the concentration was measured using BCA protein assay (ThermoScientific). 20–30 µg protein was separated on an 8–10% gel and western blot performed with antibodies as indicated in each figure. Western blot densitometry was performed by normalizing the pixel intensities of the target proteins bands with the loading control. Densitometry was carried out utilizing the analysis function provide in the Li-Cor imaging software.

### Transfection of shRNA/siRNA MCP-1

BT-549 were transfected with lentiviral particles (Origene TL316716 V) at ~ 1 multiplicity of infection (MOI). BT549 cells stably expressing MCP-1 shRNA were selected using 0.75 µg/ml puromycin (Gibco Sterile Puromycin Dihydrochloride) for 3-weeks, and then the cells were maintained in complete serum DMEM/F12 media with 0.25 µg/ml puromycin. siRNA pool for human MCP-1 (Dharmacon ON-TARGET plus L-007831-00-0005 and D-001810-10-05) and control pool were utilized to knockdown MCP-1 with Lipofectamine RNAi MAX (ThermoFisher 13778030) following manufacture’s recommendation. After 72 h the siRNA knockdown MCP-1 cells were used for experiments.

### Determining MMP9 activity by gelatin Zymography

10% Polyacrylamide gels were prepared with 1% Gelatin (final concentration) using Biorad apparatus. Cell lysate or cell-conditioned media was mixed with gel loading buffer (4 × Biorad) without DTT (Dithiothreitol) and boiling before loading. Gel separated samples were washed in 2.5% Tritron X-100 (Renaturing buffer) twice for 30 min each on a shaking platform. Renaturing buffer was then replaced with the developing buffer (50 mM TRIS, 0.2 M NaCl, 5 mM CaCl2) and gels were incubated at 37 °C for overnight. The following day, the gel was stained with Coommassie Blue R-250 for 30 min and de-stained with solution containing Methanol, Acetic acid, and Water (30: 10: 60). Picture was taken using a digital camera and processed with GIMP 2.8 on windows platform.

### Statistical analysis, and analysis and usage of publicly available data

All data presented were confirmed by at least 2 independent set of experiments. The statistical significance was determined by Student’s *t* test, and *p* < 0.05 was considered as significance. METABRIC breast cancer data were accessed using MSKCC Cbioportal (www.cbiopotal.org). The relapse-free survival (RFS) was obtained from KMPLOT (www.kmplot.com). The publicly available data were analyzed using Graph pad prism v7.

## Results

### MCP-1 expression in breast cancer cells

**Fig. 1:**
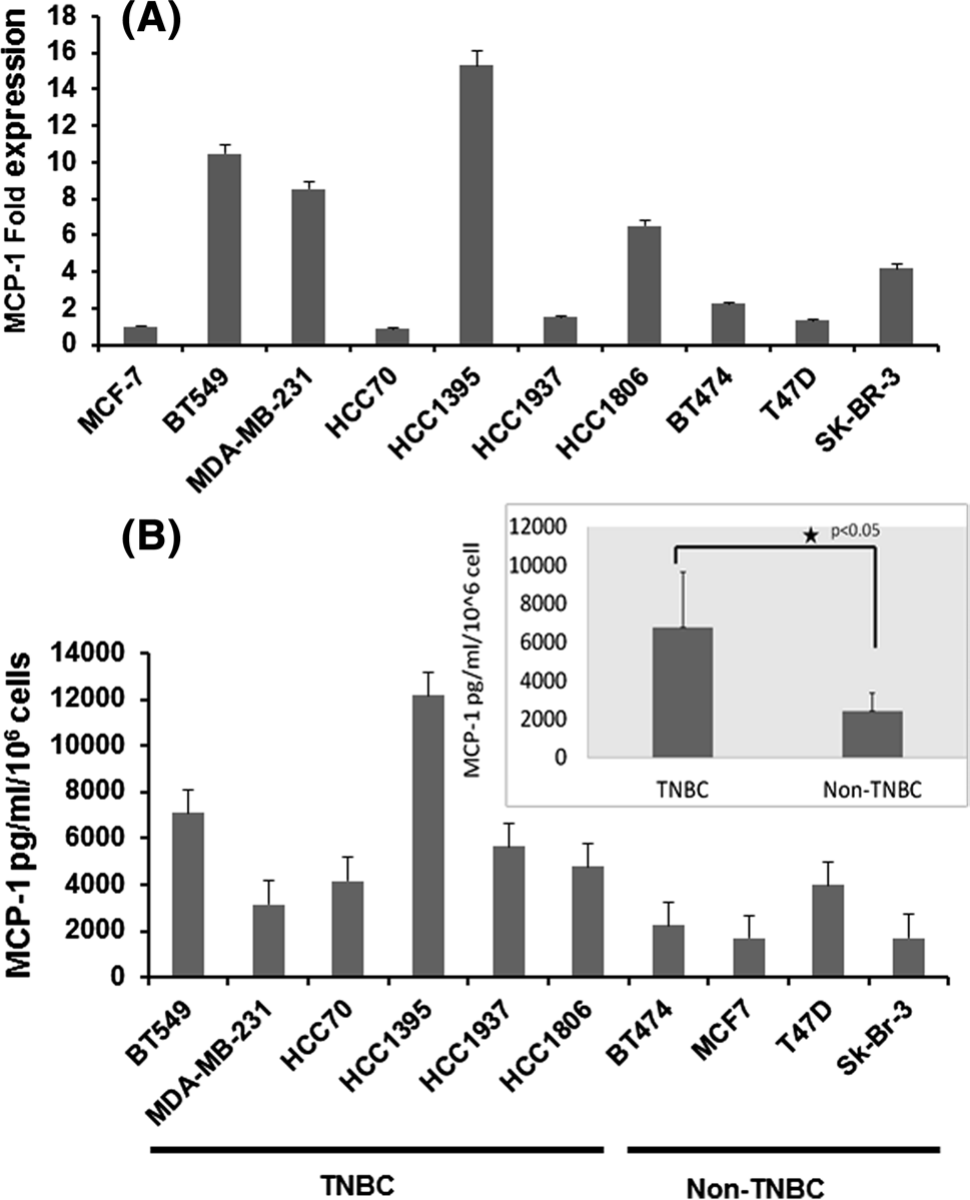
Expression of MCP-1 in breast cancer cells. **a** mRNA transcript levels of MCP-1 in breast cancer cells measured by qPCR with 18s as internal control. MCP-onefold enrichment is shown over MCF-7 luminal cell lines. RNA was isolated from the indicated cell lines in basal condition. **b** MCP-1 secretion in cell-conditioned media measured by ELISA against human MCP-1. Quantakine ELISA kit specific to human MCP-1 was used. Secreted MCP-1 levels are normalized to 10^6^ cells. MCP-1 transcript or secreted levels were measures in duplicate twice and data presented with standard deviation. *p < 0.05, *t* test

We first asked whether MCP-1 expression levels vary in different breast cancer cell type. Clinical classification of breast cancer falls into four categories based on ER, PR, and Her2 expressions as ER/PR+ and Her2−, ER/PR/Her2+, ER/PR−/Her2+, and ER/PR/Her2− (triple negative). These categories or subtypes are further categorized based on gene expression as different molecular subtypes [10–12]. The categories are Luminal A (ER/PR-positive tumor falls under this subtype); Luminal B (ER/PR/HER2-positive tumor belongs to this subtype); HER2-enriched, basal-like and mesenchymal subtypes. Triple-negative breast cancer (TNBC) is more likely to fall under basal-like or mesenchymal types [10]. In this study, we examined several of these cell types for MCP-1 expression. The TNBC/basal type cells include HCC1937, HCC11395, HCC70, and HCC1806, and the TNBC/mesenchymal-like cell lines are BT-549 and MDA-MB-231. MCF-7, BT474, and T47D were used as Luminal-type cells and SKBR3 cells represented as Her2-positive or Her2-enriched tumor type. The highest MCP-1 transcript levels were found to be in TNBC/mesenchymal type cells—BT549 and HCC1395 from our study (Fig. 1a). In addition, the secreted MCP-1 protein level was higher in most of TNBC cell lines compared with other types of cell lines (Fig. 1b).

As shown in Fig. 1b, the average secreted MCP-1 level in TNBCs was ~ 6 ng/ml/10^6^ cells, whereas it was ~ 2 ng/ml/10^6^ cells in luminal-type or receptor-positive cells (*p* < 0.05 indicated with asterisk). We also examined the canonical MCP-1 receptor CCR2 [8] in different subtypes of breast cancer cell lines, and the CCR2 expression was found to vary among those lines (Fig. S1). Luminal cell lines, MCF-7 and T47D, Her2+ line, SKBR3 and TNBC lines, BT547 and HCC1937 showed high levels of MCP-1.

### MCP-1 induces phosphorylation of p44/p42 and cell invasiveness

We first examined effect of MCP-1 in cell proliferation and the involvement of CCR2. We selected cells with low levels of MCP-1, such as MCF-7 and SKBR3, and induced the cells with recombinant human MCP-1 (rhMCP-1) followed by MTT assay. We also selected for high expression of both MCP-1 and CCR2 cell lines, such as BT549 and MDAMB-231cells. The BT549 and MDA-MB231 cells were treated with CCR2 antagonist (anti CCR2) (Calbiochem CAS 445479-97-0) followed by MTT assay. We did not find any significant effects of MCP-1 and CCR2 antagonist treatment on cell proliferation in those cells (Fig. S1A, B). Similarly, MCP-1 knockdown in BT549 cells also did not significantly change cell cycle stages as measured by propidium iodide staining (Fig. S2C). G0 stages changed to 55 from 60% after MCP-1 knockdown, possibly due to more cells being in S phage. A moderate effect was observed for S phase (18% vs. 26%) between scrambled shRNA and MCP-1 shRNA expressing cells (Fig. S2C right).

**Fig. 2:**
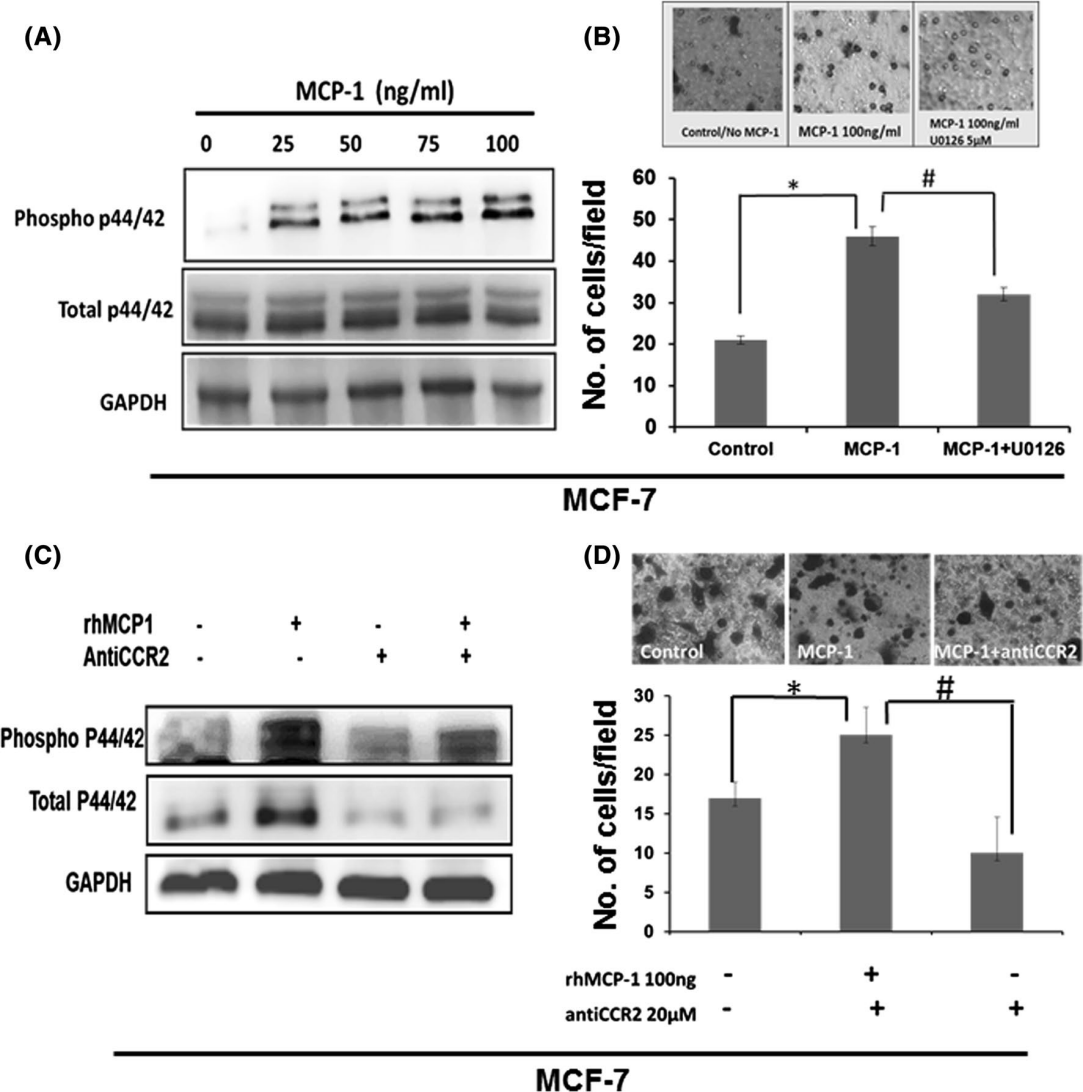
MCP-1-CCR2 axis activating the p44/42 MAP kinase and promoting cellular invasion. **a** MCP-1 treatment increases phosphorylation of p44/42 MAPK in MCF-7. Cells were serum starved overnight and treated with recombinant MCP-1 with the indicated doses, and cell lysate was prepared at 15 min time points (right). **b** Activated p44/42 MAPK is needed for MCP-1-mediated cellular invasiveness. MCF-7 cells treated with MEK inhibitor U0126 decreases cellular invasion in matrigel chamber assay. The invaded cells were counted after 48 h incubation. *N* = 3 Data shown with standard deviation, *t* test with for *p* < 0.05. **c** MCP-1 treatment results in phosphorylation of p44/42 via the CCR2 receptor. MCF-7 cells were pretreated with the 10 µM CCR2 antagonist for 2 h after 2 h of serum-free starvation. MCP-1 at the dose of 25 ng/ml was added in the presence or absence of the CCR2 antagonist. Cell lysate was prepared, and western blots were probed for phospho p44/42. **d** CCR2 is needed for MCP-1 mediated invasion in Boyden chambers invasion assay. Cells were seeded in the presence or absence of CCR2 antagonist, and MCP-1 was used as the chemoattractant. The invaded cells were counted after 38 h incubation (*N* = 3, Data shown with standard deviation, * and ^#^*p* < 0.05, *t* test, * compared between control and rhMCP-1, ^#^ compared between rhMCP-1 and rhMCP-1 with anti CCR2)

Next, we examined if MCP-1 could affect cell invasiveness. Low-expressing MCP-1 cell MCF-7 was selected and induced with rhMCP-1. It has been well known that activation of p44/42 by phosphorylation is associated with cell invasion and migration [13]. Therefore, we first examined the effect of MCP-1 on phosphorylation of MAPK. The data in Fig. 2a show phosphorylation of p44/p42 in MCF-7 cells was upregulated with the addition of 25 ng/ml rhMCP-1 for 15 min. However, 50 ng–100 ng/ml rhMCP-1 did not significantly increase the p44/p42 phosphorylation any further. The cell invasion in MCF-7 was also induced by MCP-1 for 48 h (Fig. 2b). To verify if MCP-1 cell invasion was via MAPK pathway, we treated cells with MEK inhibitor U0126, and the data showed MCP-1-induced cell invasion was significantly blocked by U0126 treatment for 48 h (Fig. 2b). Since MCF-7 expresses high level of CCR2, we also examined if blocked CCR2 could inhibit the MCP-1 induced MAPK phosphorylation and cell invasion. MCF-7 cells were treated with anti CCR2 together with or without MCP-1, and then MAPK activity was determined by Western blot analysis. The data showed that blocking CCR2 inhibited endogenous level of phosphorylation of p44/p42 as well as MCP-1-induced activation of MAPK in MCF-7 cells (Fig. 2c). As shown in Fig. 2c, the total MAPK was also inhibited by anti CCR2. MCP-1-induced cell invasion was also inhibited by anti CCR2 (Fig. 2d). The activation of p44/42 MAP kinases by MCP-1 was also observed in MDA-MB-231 cells, and this was detected using cell signaling protein array (Pathscan Array from Cell Signaling technologies) and verified with Western blot (Fig. S3A, B). As shown in Fig. S3C, the cell invasiveness of MDA-MB-231 was increased significantly upon rhMCP-1 induction, and this response was inhibited by anti CCR2. Similarly, MDA-MB-436 and MDA-MB-468 cells upon treatment of rhMCP-1 also increased cell invasiveness significantly (Fig. S4). The data suggest that MCP-1 could induce cell invasiveness via MAPK pathway without affecting cell proliferation in breast cancer cells.

### Knocking down MCP-1 inhibits phosphorylation of p44/p42 and cell invasiveness

**Fig. 3:**
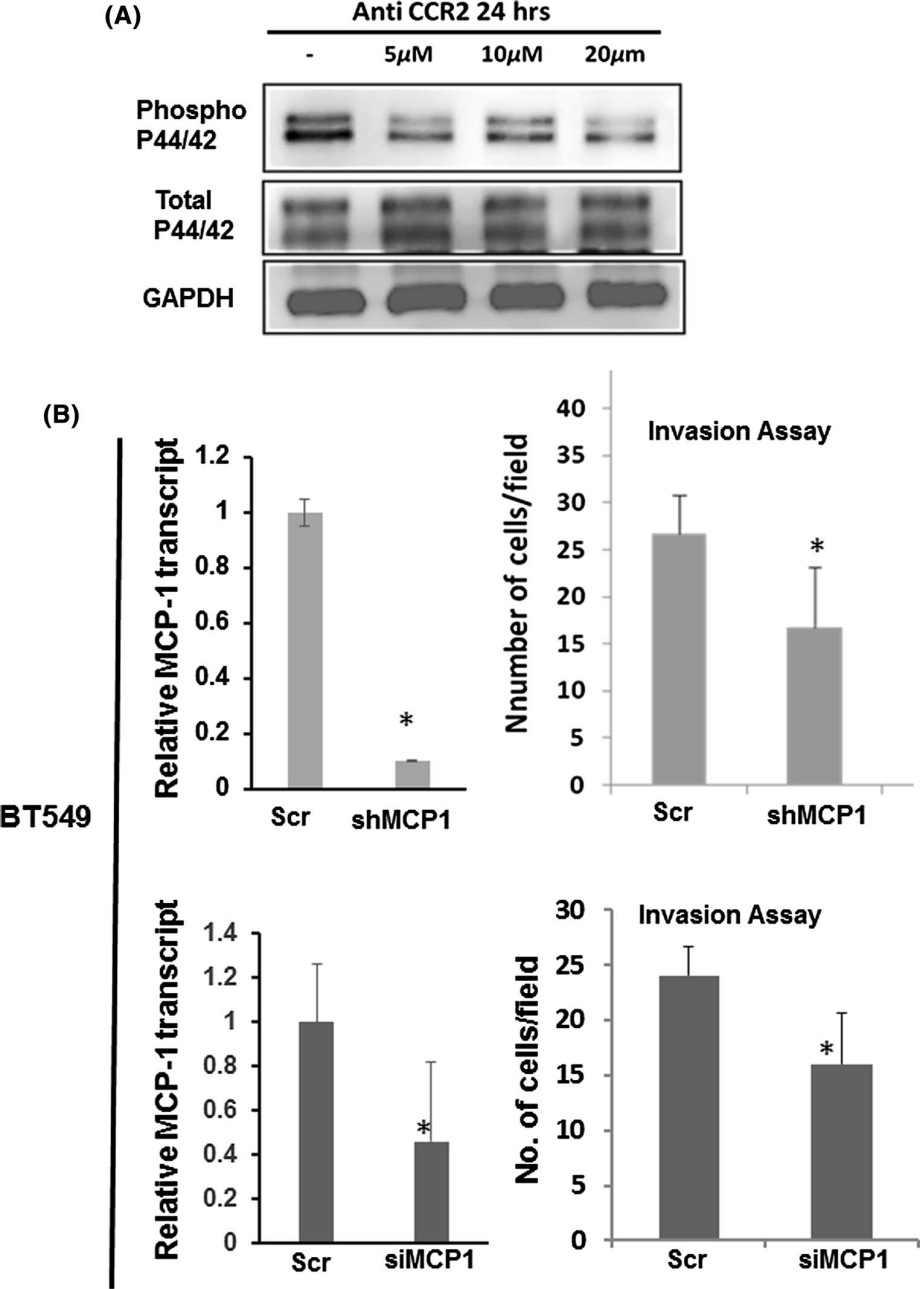
MCP-1 enhancing cellular invasiveness in triple-negative breast cancer cells. **a** MCP-1 receptor CCR2 regulates phosphor p44/42 levels in BT549 cells. BT-549 cells were treated with CCR2 antagonist at the doses mentioned. After 24 h, cell lysate was prepared, and western blots were probed for phospho p44/42. **b** Downregulating MCP-1 reduces invasion in BT549 cells. To knockdown MCP-1, BT549 cells were transfected using shRNA (top) or siRNA pool (10 nM bottom). Knockdown levels are shown for a stable line expressing shMCP1 or for the treatment with siRNA using qPCR (*N* = 2, Data with Standard Deviation, *p < 0.05, *t* test). Boyden chambers invasion assay on the scrambled control and shMCP1 shown on the right. MCP-1 knockdown cells with siRNA were also subjected to Boyden chamber invasion assay. (*N* = 2, Data shown with standard deviation, *p < 0.05, *t* test)

To further confirm the role of MCP-1 in cell invasiveness, the knocking down of MCP-1 was performed in BT549 cells. BT549 has been reported as TNBC-mesenchymal/Claudin-low type cells [14] and expresses high level of MCP-1 and CCR2 (Fig. 1 and Fig. S1). We first determined the effect of CCR2 antagonist on the phosphorylation of p44/42 levels in BT549 cells by treating the cells by increasing the concentration of CCR2 antagonist. Phosphorylation of p44/p42 in BT549 was progressively reduced followed by increasing the dosage of the CCR2 antagonist treatment, without changes in total p44/p42 (Fig. 3a). The data suggest that MCP-1 induced phosphorylation of p44/p42 via CCR2. Therefore, CCR2 could also be a potential target for inhibiting cell invasiveness in breast cancer.

Next, we knocked down (KD) MCP-1 in BT549 cells with shRNA as well as with siRNA targeting the coding region of MCP-1. Cells transfected with scrambled shRNA/siRNA were used as control. The efficiency of MCP-1 KD with shRNA and siRNA was determined by RT-qPCR first (Fig. 3b left panel) and then the MCP-1 KD BT549 cells were subjected to invasion assay. A significantly decreased cell invasion was observed in the MCP-1 KD cells compared with cells transfected with scrambled sequences (15 vs. 24–26 invaded cells per field) (Fig. 3b right panel).

### MCP-1 modulates Matrix Metalloprotease 9 (MMP9) and EMT associated protein in breast cancer

**Fig. 4:**
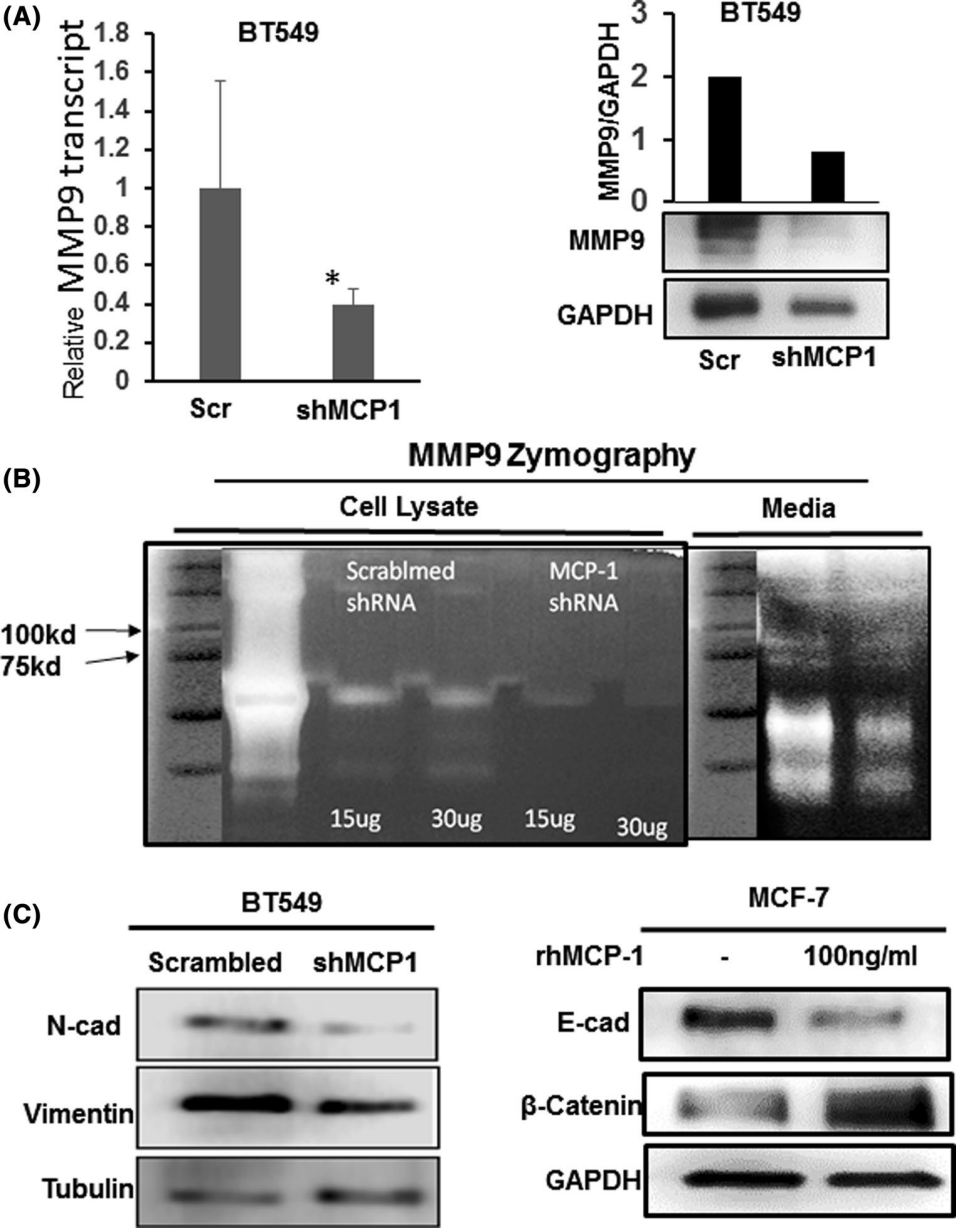
MCP-1 affecting breast cancer invasiveness via MMP9 and EMT associated proteins. **a** MMP9 transcript levels in BT549 cells expressing MCP-1 shRNA or scrambled control. MMP9 mRNA level in shMCP-1 cells is shown with respect to control by qPCR, *N* = 2, Data with Standard Deviation, *p < 0.05, *t* test. MMP9 protein levels are shown on the right in the control and knockdown cells. **b** MMP9 activity in BT549 cells upon MCP-1 knockdown. Gelatin zymography shown for cell lysate from control and shMCP1 BT549 cells on the left. On the right is cell-conditioned media collected from the same cells. Proteins were separated on 10% gel containing 1% gelatin as substrate for MMP, as described in material Method. **c** EMT markers N-cadherin and Vimentin are shown in BT-549 cells expressing MCP-1 shRNA by western blot (left) on cell lysate prepared from shMCP-1 and scrambled control. E-cad and β-Catenin levels are shown for 100 ng/ml rhMCP-1 treated MCF-7 cells at 1 h relative to untreated control. Tubulin or GAPDH is shown as the loading control

MMP activity is associated with cancer metastasis, as secreted MMPs help cancer cells to extravagate by digesting extracellular matrix [15]. Interestingly, MMP9 has been implicated TNBC cells invasiveness. Therefore, we tested whether knocking down MCP-1 also affected the levels of MMP9. Our data showed that MMP9 transcripts in shMCP-1 BT549 cells were downregulated (Fig. 4a left). Accordingly, MMP9 protein level was also reduced in shMCP-1 BT549 cell lysate (Fig. 4a right). The data suggest that higher activity of MMP9 may be involved in matrix digestion in the cells. Next, we examined MMP9 activity by gelatin zymography in cell lysate and in condition media. The data in Fig. 4b showed that shRNA knockdown MCP-1 reduced activity of MMP9 in both cell lysate and condition media.

It is known that cellular invasiveness is associated with gain of mesenchymal phenotype and loss of epithelial morphology via a process called epithelial-to-mesenchymal transition (EMT). At the molecular level, cancer cells showing higher metastatic potential go through the process of EMT prior to metastases [16]. Therefore, we also examined role of MCP-1 in EMT. We have observed knockdown MCP-1 in BT549 cells reduced N-cadherin and Vimentin, and when MCF-7 cells were treated with rhMCP-1, it downregulated E-cadherin and upregulated β-catenin (Fig. 4c). Those markers are well known as EMT-associated markers [17]. However, we did not observe any morphological changes or upregulation of EMT-related transcription factors in MCF-7 cells upon MCP-1 treatment or BT549 cells upon knockdown. Experiments with longer period of time treatment may be required for further validation.

### MCP-1 is highly expressed in patients with Claudin-low and basal types breast cancer

**Fig. 5:**
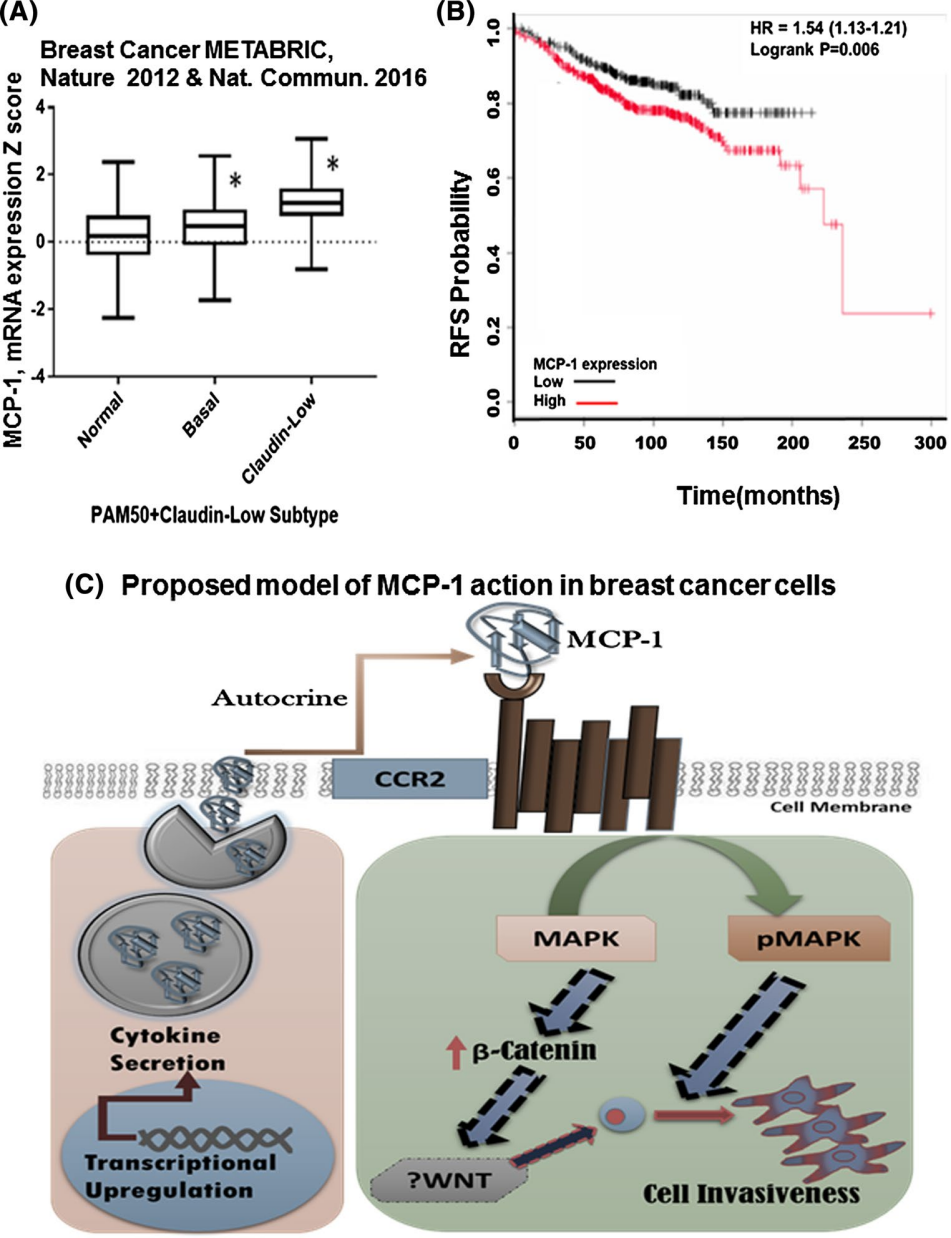
Highly expressed MCP-1 in patients with basal and claudin-low type breast cancers. **a** METABRIC dataset showing expression of MCP-1 in Basal and Claudin-low type breast cancers, which are predominantly TNBC type of breast cancer. mRNA expression in cancer tissue shown for Affymetrix U133A array data. **b** Relapse-free survival (RFS) probability in breast cancer patient with basal type intrinsic subtype (kmplot.com). **d** Proposed model of MCP-1 action in breast cancer cells. Upregulation of MCP-1 activates MAP kinase (p44/42) via activation of the CCR2 receptor on the breast cancer cell surface. Upregulation of β-catenin can also contribute to cell invasion by possibly upregulating WNT TNBC cells shows higher expression of MCP-1, and can therefore be affected by MCP-1 to be more invasive

We have shown that MCP-1 expression is higher in breast cancer cell lines that are predominantly TNBC type (Fig. 1). Since several of these TNBC cells belong to Basal type and Claudin-low type cancers, we queried the METABRIC breast cancer dataset [18] for MCP-1 expression in those two subtypes via Cbioportal [19]. Both basal and Claudin-low subtype breast cancer tissues showed significantly higher expression of MCP-1 mRNA in cancer tissues (Fig. 5a). To explore whether MCP-1 expression would have prognostic value, we also utilized data from KMPLOT [20] and queried relapse-free survival (RFS) in basal type breast cancer patients with high or low MCP-1 expression. The MCP-1 expression was measured by microarray in the dataset, and expression levels 1.5 times higher than normal or noncancer tissue was used for assessing RFS. The data showed that RFS was significantly lower in patients with higher expression of MCP-1 (red line, median split) compared to those with lower expression of MCP-1 (black line) (Fig. 5b).

## Discussion

A heterogeneous disease like breast cancer can manifest in various different clinical pathological subtypes. Among these different subtypes, TNBCs pose a challenge to breast cancer therapy because of its lack of identified target and propensity to relapse. We have shown that MCP-1 is upregulated in triple-negative breast cancer and can serve as a prognostic/diagnostic marker for TNBC cells. Interestingly, MCP-1 expression in individuals with breast cancer differ depending on the disease specific subtype [7]. Her2-positive and TNBC (basal type) breast cancer cells show higher expression of MCP-1 than ER/PR-positive breast cancer cells (Fig. 5). Interestingly, MCP-1 has been shown to cooperate with RANTES to promote angiogenesis in breast cancer patients [21]. Thus, MCP-1 is possibly a part of a chemokine network that regulates breast tumorigenesis by augmenting inflammatory milieu. Inflammation in breast cancer is associated with poor outcome for patients, with advanced stage of the disease. In triple-negative breast cancer, inflammation plays an important role in modifying Immune environment. Inflammation usually is more permissive for the cancer to grow and disseminate to distant organs.

In our work, we have shown, that MCP-1 upregulation can enhance cell invasion. In breast cancer cells, MCP-1 mediated its effect via the canonical CCR2 receptor in an autocrine manner (Fig. 5c model). Our data also indicate that MCP-1 mediated cellular invasion works via the MAP kinase pathway. Since, MCP-1 is able to upregulate β-Catenin in MCF-7 cells, we posit that MCP-1 can also contribute to cell invasion by modifying Wnt in breast cancer cells (Fig. 5c). Interestingly, It has been shown that β-Catenin can transactivate MCP-1 promoter in breast cancer cells [22] Thus, a positive feedback loop may be able to transcriptionally upregulate MCP-1 in breast cancer. Our data provide evidence for MCP-1 to be a prognostic marker for triple-negative breast cancer. Our findings could address health disparity in this area by opening up specific treatment avenues for patients with higher expression of MCP-1. MCP-1 antibody (ABN912) has been used in clinical trial for rheumatoid arthritis. However, the antibody therapy failed to show any clinical and immunological improvement with respect to placebo [23]. It would be interesting to see whether other antibodies could be a therapeutic option for women with TNBC, who also exhibit high MCP-1 levels. To date, the primary treatment option for TNBC is chemotherapy [2] and MCP-1 has been shown to protect breast cancer cells from apoptosis following treatment with chemotherapeutic agents [24]. Hence, MCP-1 antibodies or CCR2 receptor antagonist in combination with chemotherapy might be able to achieve pathologic complete response (pCR) as TNBCs are known to respond well to neoadjuvant chemotherapy. However, if pCR is not achieved, TNBCs tend to relapse more frequently compared to luminal A or B type breast cancer (TNBC paradox) [25]. MCP-1 could be one of the key factors in this phenomenon. This is because, chemotherapeutics are known to induce inflammatory cytokine like MCP-1 [26]. Higher MCP-1 expression/secretion following neoadjuvant chemotherapy can conceivably contribute to relapse as well as metastasis [27].

MCP-1 is also well known in macrophage-related migration and has been shown to be involved in breast cancer progression [28]. MCP-1 is one of the cytokines secreted from adipocytes that may precipitate inflammation. Both macrophages and adipocytes can contribute to tumor microenvironment that permits tumor growth [29]. On the other hand, higher expression as well as secretion MCP-1 from TNBC cells can recruit macrophages in the tumor environment in vivo. Tumor associated macrophage show an M2 phenotype, which helps the tumor to grow. In recent years, macrophages have been shown to aid tumor cells to extravasate and metastasize. Thus, higher levels of MCP-1, in the tumor microenvironment, can be instrumental in enhancing cancer cell invasiveness by activating both autocrine and paracrine pathways [30]. Further work will be necessary to understand whether MCP-1 secreted from cancer cells and cells in the tumor micro environment (macrophages, adipocytes, and fibroblast) will have different roles to play in terms to tumor initiation and maintenance.

Our data, thus far, indicates that higher MCP-1 level is associated with TNBC and has the potential to affect cell invasion by activating MAP kinase pathway in an autocrine manner. In accordance with that, we have found that higher MCP-1 levels in basal type breast cancer lead to lower survival in patient. TNBC is also a disease with significant health disparity. Younger African-American women have higher incidence of TNBC, resulting in increased mortality. Our findings could address this health disparity by opening up specific treatment avenues for patients with higher expression of MCP-1. It would be interesting to see whether MCP-1 antibodies could be a therapeutic option for women with TNBC with high MCP-1 levels. For example, MCP-1 antibody (ABN912) has been used in clinical trial for rheumatoid arthritis without much success [23]. This antibody can potentially be used to treat TNBC patients instead. Thus, the prognostic value of our finding can be utilized to design better therapies for TNBC patients with basal or mesenchymal type of gene signature associated with the disease.

## Electronic supplementary material

Below is the link to the electronic supplementary material.

Supplementary material 1 (DOC 28 kb)

Supplementary material 2 (DOCX 12 kb)

Supplementary material 3 (TIFF 132 kb)

Supplementary material 4 (TIFF 189 kb)

Supplementary material 5 (TIFF 200 kb)

Supplementary material 6 (TIFF 184 kb)
