# Supplementary material for: MCP-1 is overexpressed in triple-negative breast cancers and drives cancer invasiveness and metastasis
Source: Breast Cancer Res Treat. 2018 Mar 28;170(3):477–86. doi: 10.1007/s10549-018-4760-8 (PMC6022526; doi:10.1007/s10549-018-4760-8)
Supplement: Supplementary file 1 — Supplementary material 1 (DOC 28 kb) [file 10549_2018_4760_MOESM1_ESM.doc]

**Supplementary Materials and Methods:**

**Antibodies and reagents:**

The following reagents were used for the study: CCR2 Antagonist - CAS 445479-97-0 from Calbiochem. Recombinant human MCP-1, 279-MC-010 from R&D Systems, MCP-1/ CCL2 (Gene ID 6347) Human shRNA (TL316716V) and scrambled shRNA from Origene. Optimem Reduced Serum medium (Invitrogen), Human CCL2/MCP-1 Quantikine ELISA Kit (DCP00) from R&D Systems, Quantabio first strand cDNA synthesis super-mix (Catalogue 95048 -100), Quantitect SYBR Green qPCR Mix (Qiagen). Other reagent are as follows: Thiazolyl Blue Tetrazolium Bromide (MTT) from Sigma; MEK inhibitor U0126 (Cell Signaling), E-Cadherin, N-Cadherin and Vimentin antibody (Epithelial-Mesenchymal Transition (EMT) Antibody Sampler Kit 9782, Cell Signaling), phospho-p44/42 (ERK1/2 Thr202/Tyr204) and total p44/42 antibody (Cell Signaling), MAPK antibody (Cell Signaling), β-actin and GAPDH mouse antibody (Santa Cruz), PathScan® RTK Signaling Antibody Array Kit (Cell Signaling Technologies 7982).

**Primers’ Sequences used for quantitative real-time PCR:**

MCP1-Forward: GGCTGAGACTAACCCAGAAAC MCP1-Reverse: GAATGAAGGTGGCTGCTATGA

18S – Forward: gatccattggagggcaagtc 18S – Reverse: tcccaagatccaactacgag

MMP9 primers were from RTPrimerDB ([www.rtprimerdb.org/](http://www.rtprimerdb.org/)) Id 983

MMP9- Forward: GTGCTGGGCTGCTGCTTTGCTG

MMP9_ Reverse: GTCGCCCTCAAAGGTTTGGAAT

**Cell proliferation assay/Cell cycle analysis:**

For MDA-MB-231 and BT549 cell, 3000 cells/well were plated in each well of a 96-well plate and assessed for growth inhibition with CCR2 antagonist. After 72 hrs. of treatment, cell proliferation rates were determined using MTT assay as per manufacturer’s protocol. The media from the plates were discarded and 50 µl of MTT reagent was added to each well. After incubation with MTT for 2 h at 37^o^C, the reagent was discarded and 100µl DMSO was added. The plates were then placed on a shaker for 15min at RT. The absorbance was read at 560 nm. Experiments were performed in quadruplicate. The results are presented as percentage of cell proliferation with respect to untreated cells. Conversely, for cell proliferation with human recombinant MCP-1 (rhMCP-1), 5000 MCF7 and SK-BR-3 cells were plated in 96-well plates in phenol-red free media with 1% serum. Indicated concentration was added the following day, with BSA as stabilizing reagent. MTT assay was performed as before after 72 hours incubation.

Cell cycle analysis was performed with 70% ethanol fixed BT549 cells expressing scrambled or MCP-1 shRNA. Fixed cells were stained with FxCycle PI/RNase Staining Solution (ThermoScientific F10797) and Data captured with Attune flow cytometer.
