# Supplementary material for: MCP-1 is overexpressed in triple-negative breast cancers and drives cancer invasiveness and metastasis
Source: Breast Cancer Res Treat. 2018 Mar 28;170(3):477–86. doi: 10.1007/s10549-018-4760-8 (PMC6022526; doi:10.1007/s10549-018-4760-8)
Supplement: Supplementary file 2 — Supplementary material 2 (DOCX 12 kb) [file 10549_2018_4760_MOESM2_ESM.docx]

**Supplemental Figure Legends:**

**Figure S1. MCP-1 canonical pathway receptor CCR2 levels in the breast cancer cells.** The level of CCR2 expression was determined using western blot with antibody against CCR2 receptor. GAPDH is used as loading control (bottom panel). Western blots were conducted twice with two independently derived cell lysates. The gels were quantified with the software provided in analysis function of the Li-Cor imaging system. The bars in top panel indicated mean level of CCR2 protein from the duplicated western blots and standard deviation.

**Figure S2.** **MCP-1 and CCR2 do not affect cell proliferation.** TNBC cells MDA-MB-231 and BT-549 were treated with CCR2 antagonist at the indicated concentrations (A) and MCF-7 and SKBR3 cells were treated with rhMPC-1 (B). MTT assay was performed to assess cell proliferation after 72 hrs treatment. The bar graphs indicated percentage of cell growth. **(C)** Cell Cycle analysis with propidium iodide. Different cell Cycle stages were shown for scramble control and MCP-1 knockdown cells.

**Figure S3. MCP-1 activates the MAP Kinase pathway**. (A) Serum starved MDA-MB-231 cells were stimulated with recombinant human MCP-1. Cell lysates were probed on a PathScan RTK Signaling Array Kit from cell signaling technologies. (B) Western blot was performed to verify phosphorylation of p44/42 (Thr202/Tyr204) upon MCP-1 treatment. Indicated doses of MCP-1 was added after overnight serum starvation (0.5% Serum) on MDA-MB-231 cells. Cell lysate were prepared after 30mins of addition rhMCP-1 and western blot performed as before. (C) Boyden Chambers invasion assay with triple negative MDA-MB-231 cells. Cells were seeded in the presence or MCP-1 and in combination with anti CCR2. (N=3, Data shown with Standard Deviation, * and # p<0.05, t-test, * compared with untreated control, # compared between rhMCP-1 and rhMCP-1 with anti CCR2).

**Figure S4.** **MCP-1 mediated Boyden Chamber invasion assay**. (A) mRNA levels of MCP-1 in MDA-MB-436, MDA-MB-468 and MCF-1 cells were determined by RT-qPCR. (B) MDA-MB-436 and MDA-MB-468 cells were treated with rhMCP-1 and cell invasion was determined by Boyden Chamber Invasion Assay. The invaded cells were counter after 24 hours. The bars indicated mean of invaded cells from two time’s independent experiments with Standard Deviation shown. * p<0.05 tested by t-test.
