# Supplementary figures and images for: MCP-1 is overexpressed in triple-negative breast cancers and drives cancer invasiveness and metastasis
Source: Breast Cancer Res Treat. 2018 Mar 28;170(3):477–86. doi: 10.1007/s10549-018-4760-8 (PMC6022526; doi:10.1007/s10549-018-4760-8)

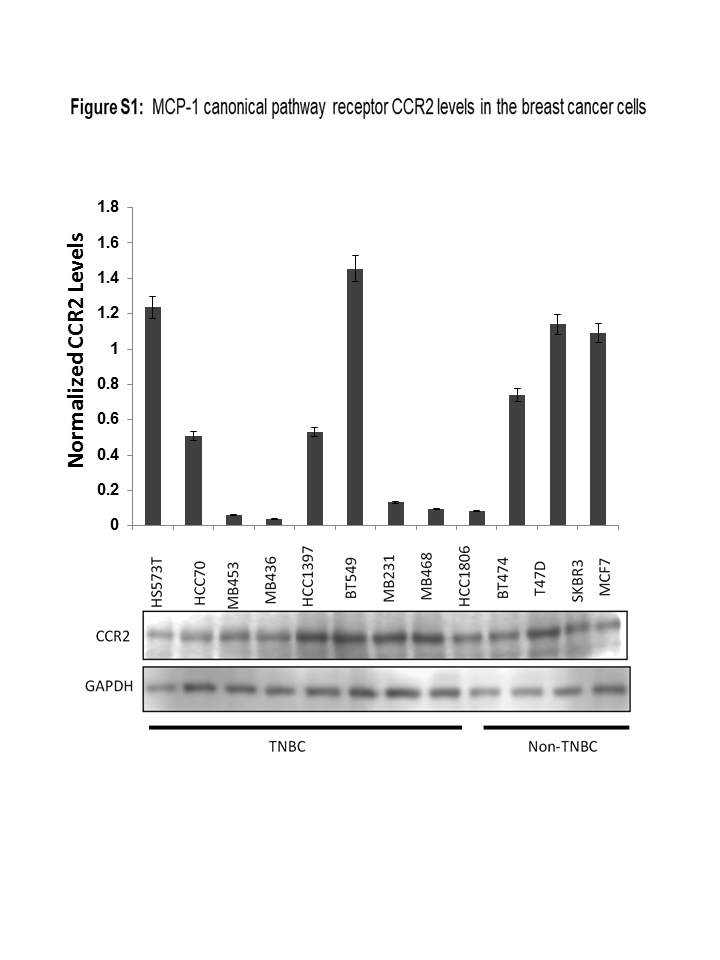

Supplement: Supplementary file 3 — Supplementary material 3 (TIFF 132 kb) [file 10549_2018_4760_MOESM3_ESM.tif]

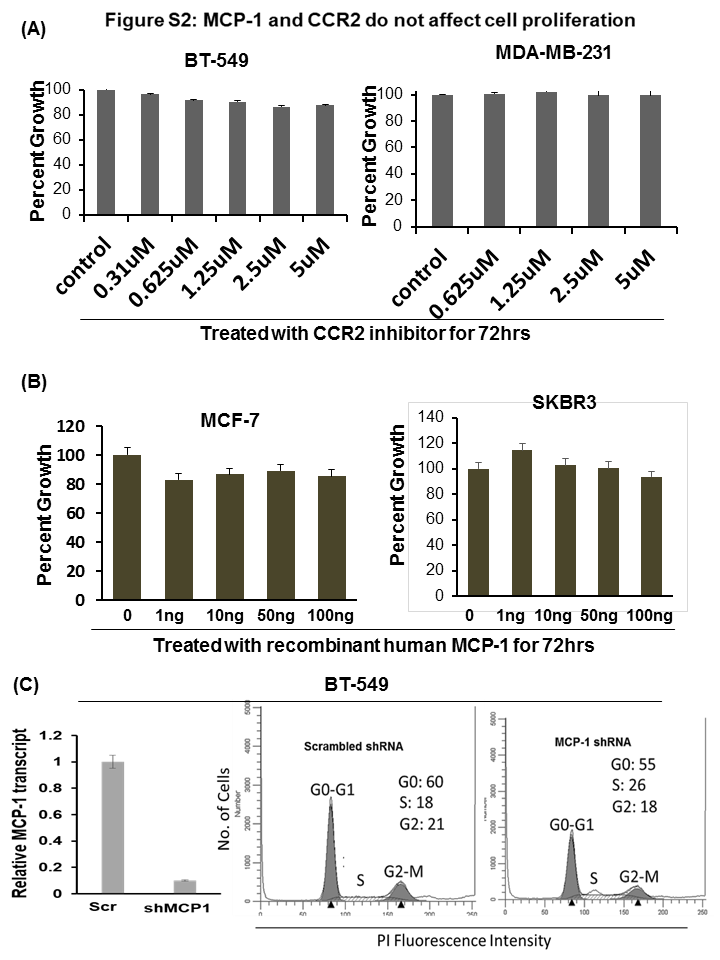

Supplement: Supplementary file 4 — Supplementary material 4 (TIFF 189 kb) [file 10549_2018_4760_MOESM4_ESM.tif]

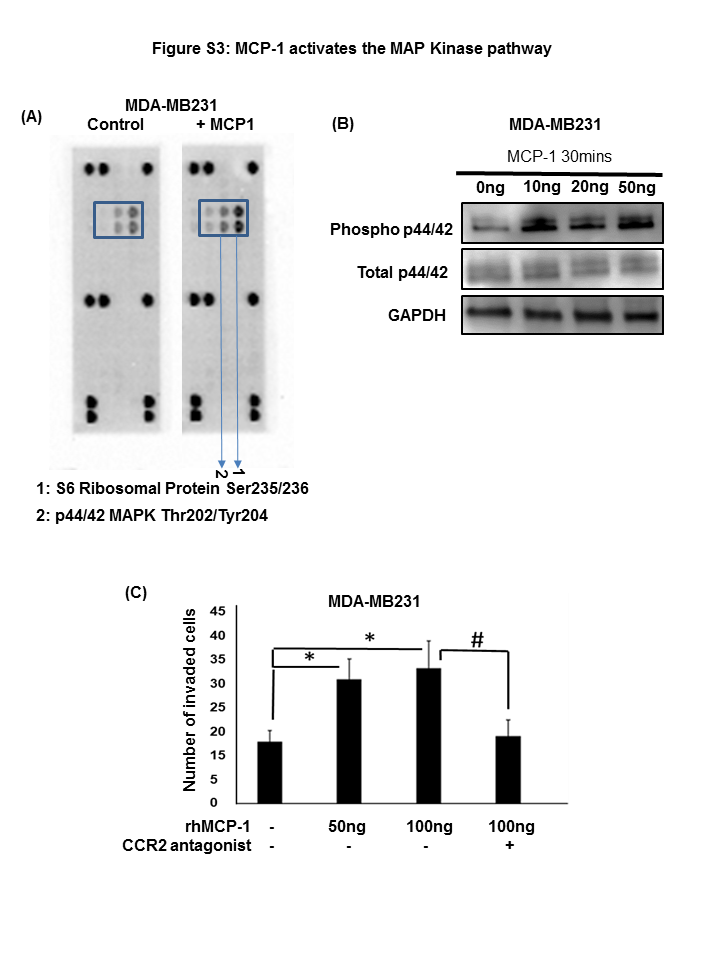

Supplement: Supplementary file 5 — Supplementary material 5 (TIFF 200 kb) [file 10549_2018_4760_MOESM5_ESM.tif]

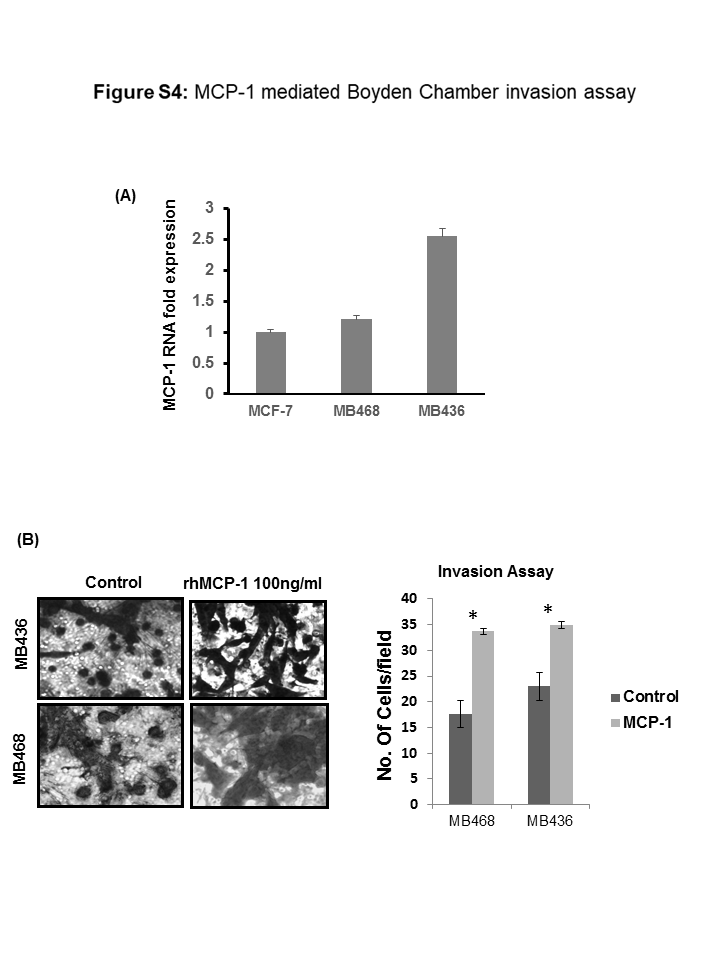

Supplement: Supplementary file 6 — Supplementary material 6 (TIFF 184 kb) [file 10549_2018_4760_MOESM6_ESM.tif]
